# COMPANIONSHIP TO ADDRESS QUALITY OF LIFE AND LONELINESS AMONG OLDER ADULTS WITH SEVERE LONELINESS

**DOI:** 10.1093/geroni/igac059.2609

**Published:** 2022-12-20

**Authors:** Kelsey McNamara, Ellen Rudy

**Affiliations:** Papa Inc., Miami, Florida, United States; Papa Inc., Miami, Florida, United States

## Abstract

Research indicates that loneliness is a powerful predictor of premature mortality. Less is known about those who experience “severe loneliness” and their responsiveness to companionship as a means to clinically improve. We sought to understand the epidemiology of a cohort of lonely older adults and the impact of companion care on severe loneliness and quality of life. Companion care was provided through Papa Inc., a national service that pairs older adults with “Papa Pals” who provide companionship and assistance with everyday tasks. Participants have free access if their Medicare Advantage plan offers it. The sample included adults ages 65+, active in Papa in 2021, and provided follow-up data (UCLA-3 Loneliness Scale; CDC's Healthy Days Measures) as of September 2021. Analysis utilized t-tests and Chi-square tests with significance set at p< 0.05. A total of 2650 participants were identified as lonely at baseline, 435 were successfully contacted to collect complete follow-up data. Of the follow-up cohort, 22% were classified as severely lonely at baseline reporting mean UCLA score of 8, and 15 mentally and 14 physically unhealthy days on average. Of those severely lonely, 62% were female, 11% were ages 85+. Sixty percent of severely lonely participants experienced clinical improvements and moved to a lower category of loneliness (mean change in UCLA: –3.37) and reduced their unhealthy days (–6.20 mental; –2.09 physical). This real-world evidence suggests a companionship program can improve loneliness and quality of life among severely lonely older adults. Earlier interventions to prevent chronic loneliness should be explored.

